# Tomato flu 2022 outbreak in India: Issues, challenges, and possible solutions

**DOI:** 10.1002/hsr2.1174

**Published:** 2023-04-01

**Authors:** Ridwan Olamilekan Adesola, Ibrahim Idris, Emmanuel Opuni, Joanna Nicole D'Souza

**Affiliations:** ^1^ Department of Veterinary Medicine, Faculty of Veterinary Medicine University of Ibadan Ibadan Nigeria; ^2^ Department of Veterinary Medicine, Faculty of Veterinary Medicine Usmanu Danfodiyo University Sokoto Nigeria; ^3^ Department of Health Policy The London School of Economics and Political Science London UK; ^4^ Department of Biotechnology R.V. College of Engineering Bangalore India

**Keywords:** children, India, Tomato flu

## Abstract

**Background and Aims:**

The outbreak of Tomato flu in India has affected the children making the parents tremble in worry and pain. The outbreak of this disease first occurred in India affecting young children below 5 years of age, thereby causing a threat to the country, neighboring countries, and the rest of the world—although no death is yet recorded. The aim of this research is to discuss the issues, challenges, and possible solutions associated with 2022 Tomato flu outbreaks in India.

**Results:**

Tomato flu is caused by Coxsackievirus A16 as confirmed in the United Kingdom. The health authorities are currently monitoring the spread of the virus and trying to understand it to develop strategies to contain it. But they are left with some challenges relating to the health system, surveillance, and adherence to preventive protocols among others.

**Conclusion:**

To prevent the spread of Tomato flu to other neighboring countries like China, Bangladesh, Pakistan, Sri Lanka, Myanmar, Afghanistan, Bhutan, Nepal, and the Maldives, the Indian Government must provide adequate public health control measures to contain the disease in children. Various recommendations have been given below.

## 1 INTRODUCTION

In the 21st century, infectious diseases are emerging and evolving. Three years ago, precisely in 2019, the first outbreak of coronavirus disease occurred in China, which metamorphosed into a pandemic unprecedented in the 21st century.^1^ It is still a threat to global health, although efforts are currently in place to avert its spread and transmission; vaccines for prevention and drugs for treatment have been developed. Earlier in 2022, there was a Monkeypox outbreak in the United Kingdom,^2^ which later spread to other European countries, a Langya virus outbreak in China,^3^ a Marburg virus disease outbreak in Ghana, in Africa,^4^ and presently a Tomato flu outbreak in India.^5^

Tomato flu is a disease characterized by rashes and flu‐like symptoms spreading among children.^5^ Initially, there was confusion about the etiology of the disease, but it has been confirmed as an enterovirus disease in the United Kingdom.^6^ There are still ongoing studies to characterize the virus in India. The disease mostly affects children below the age of 5. Tomato flu disease in India poses a health threat to children in India, the neighboring countries, and over the globe—though no deaths have been recorded yet due to its mode of transmission, and novelty among others. Children are the leaders of tomorrow,^7^ the emergence of Tomato flu poses a significant burden on the population growth of children in India. The virus has minimum potential to become a pandemic due to its low virulent ability, alarming levels of spread, and alarming stages of inactions. Although, if adequate biosecurity measures are not put in place, it might cause a great alarming public challenge to the children.

Tomato flu is caused by Coxsackievirus A16 (CA16) as confirmed in the United Kingdom.^6^ Coxsackieviruses are a few closely related enteroviruses that belong to the family Picornaviridae of nonenveloped, linear, positive‐sense single‐stranded RNA viruses.^8^ Apart from Coxsackieviruses, enteroviruses include the subgroups echoviruses, and polioviruses. Every year, there are thought to be 1 billion or more enteroviral infections worldwide. With coxsackievirus A6, echovirus 11, echovirus 18, coxsackievirus A9, coxsackievirus B4, echovirus 30, echovirus 6, and enterovirus D69 accounting for over 50% of enteroviral infections in the United States each year, there are roughly 10–15 million symptomatic cases.^8^

CA16 is one of the major pathogens associated with hand, foot, and mouth disease (HFMD) in infants and young children. Although scientists are still trying to understand the pathogen. The infection produced an eruption of red blisters which later grow and transformed to tomato size, given the name Tomato flu.^5^ The main clinical signs of Tomato flu detected are almost indistinguishable from that of chikungunya, these include joint pain, increased body temperature (high fever), and rashes.^9^ These rashes also result in skin irritation, other symptoms include vomiting, diarrhea, dehydration, muscle pain, and arthritis.^10^ Tomato flu is an endemic viral infection in India but is underreported due to poor disease surveillance in India, where children of young age, specifically those below 5 years of age are the most susceptible to the disease.^11^ Infection is possible among humans by direct contact with the infectious virus, which is present in the saliva, blister fluid, nose and throat secretions, and stool of those who are infected. The virus is usually transferred by contact with surfaces that have been exposed to the virus.^12^ Although patients naturally recover, even without medication, between 7 and 10 days, it is highly infectious even after symptoms have disappeared.^12^ As a novel type of HFMD, there is no specific treatment for it, but like other flu diseases antipyretics and analgesics and usually prescribed, accordingly. The disease is evolving, and it is also being researched to understand its origin, biology, and control.^11^, ^12^

## 2 ACCOUNT OF THE OUTBREAK

The first case of Tomato flu, also known as Tomato fever, was identified in May 2022 in the Kollam region of Kerala, India. The index case is a 4‐year‐old child from a village near the Kerala–Tamil Nadu border, which was reported on May 6, 2022.^12^ Subsequent cases have been reported in Tamil Nadu and the Odisha states in India and the United Kingdom.^6^, ^12^ After 2 months, more than 80 young children below 5 years and even adults with weaker immunity become infected.^12^

## 3 PUBLIC HEALTH RESPONSE

Health authorities have started different public health measures to contain the disease. For example, some children's health centers have been closed to avoid infections from the clinics, and a disease awareness program has also started.^12^ Furthermore, the Uttar Pradesh government shared an advisory communication with all the Chief Medical Officers of 75 districts in New Delhi that parents should inform their children not to hug other children with rashes and headaches. In Coimbatore, disease tests are being carried out at Anganwadi centers, and 24 mobile teams with medical personnel have reportedly been sent out. To keep an eye out for anyone with a fever and rashes, three teams of tax inspectors, health inspectors, and police have been sent out in shifts. Since Kerala accounts for most of the cases, the surrounding areas are closely monitored. All hospitals in the neighboring states' outpatient departments have been told to report any patients exhibiting Tomato flu symptoms. The neighboring state of Tamil Nadu has enhanced border surveillance in response to cases of Tomato flu being found in Kerala. The Kerala government launched initiatives in each Anganwadi and medical facility to treat the affected people.^13^

## 4 HEALTH SECURITY ISSUES AND CHALLENGES

Although no death related to Tomato flu has been reported, it poses a health security threat due to the nature of the disease, the ages mostly affected, India's huge population, and the high possibility of spreading to other countries (if proper biosecurity measures are not put in place). The disease mostly attacks children, and it is difficult to control disease among children under 5 due to their inability to understand and follow preventive procedures, causing a higher probability of spreading faster. Again, India has a huge population, therefore, many people can be affected if the control measures are not adhered to well. The disease can also spread to other countries if the disease is not controlled swiftly due to international travel to and from India. Two cases have already been reported in the United Kingdom^10^, ^14^ and it can spread to other countries due to international travel to and from the United Kingdom. There are a couple of challenges facing the Indian health system in fighting Tomato flu.

### 4.1 Health infrastructural issues

The biggest healthcare problem facing India's health industry right now is undoubtedly a lack of infrastructure. The nation's healthcare system is always under strain due to a lack of hospital beds, a shortage of specialists to handle serious illnesses, and excessive out‐of‐pocket costs. The pressure on this faltering healthcare system is now hanging by a thread, especially given the low rate of professional training compared with other nations. This contributes negatively to the response of healthcare professionals to Tomato flu eradication.

### 4.2 Affected age group

The impact of this disease is on children under 5 years. It is very difficult to get children at this age to adhere to preventive measures to the letter and it is not different in India. Hence, the disease can spread easily among children leading to a challenge in controlling the disease. The disease can spread to other states in India, neighboring countries, and the rest of the world because of travel, trade, religious and social gatherings, and so forth. Therefore, this is a great burden not only to the government and citizens of India but to the neighboring countries, and the world. Although, the virus has minimum potential to become a pandemic due to its low virulent ability, alarming levels of spread, and alarming stages of inaction.

### 4.3 Large number of populations

The large population of Indians can be another challenge in controlling the outbreak. Longer life spans as well as a growing global population place a heavy burden on India's healthcare infrastructure. India is the second most populous nation in the world so most infectious diseases spread quite quickly usually due to the density of the population coupled with poor hygiene practices among others. As Indians gather for social and religious events, interactions increase, the risk of this infection among children is highly possible, and religious and social events have not been restricted. Therefore, it is difficult to control the disease with children and big populations interacting in religious and social events.

### 4.4 Poor health insurance schemes

There is widespread evidence that Indian households suffer catastrophic health expenditures.^15^ Community health insurance systems in India are barely successful^16^ and only 3% of the population is covered by efficient health insurance schemes.^14^ Also, it was quite challenging for the average person to acquire the full scope of in‐patient care for Tomato fever due to a lack of knowledge about healthcare insurance. Following these, Tomato flu is likely to cause a financial burden which can lead to households trying to treat the disease at home, thereby creating difficulty in fighting the disease.

### 4.5 Child mortality rates

Despite the incredible technological improvements made over the past 20 years, India's death rates continue to be high while the rest of the globe is experiencing a reduction. India has one of the highest infant mortality rates in the world at 721,000, according to an article published by Gill et al.^17^ There could possibility of a misconception about this mortality rate for Tomato flu since Tomato flu is also affecting young children. The idea that prenatal care and surgical procedures are too expensive to be handled by the family is one of the main causes of ongoing death rates. Making people aware of inexpensive interventions that are just as effective as more expensive ones is what needs to be done. Long‐term mortality rates will be decreased as a result, and the public's confidence in Indian healthcare will also rise.

### 4.6 Unmanageable patient load

Providing healthcare for 1.4 billion people,^18^ or around 20% of the world's population, is a massive effort in and of itself. It should be everyone's top priority to manage medical and human resources sustainably to meet the needs of the next generation. Healthcare facilities should use technology whenever possible to improve operational and clinical processes to drive an adequate patient flow. Also, it might be challenging to promote telehealth services and virtual care practices, both of which can greatly lessen patient load stress.

### 4.7 Mode of transmission of the disease

The risk of transmission through the mother is unavoidable means of transmission of the disease. The disease easily transmits from mothers to newborn babies, and this could be the most serious.^12^ Unfortunately, the mother does not show symptoms of the disease when they are infected making it difficult to control the spread of the disease to children. Typical of HFMD, infected people usually show no symptoms of the disease in the 1st week, but they are contagious around this time.^12^ This makes it difficult to fight the disease without the involvement of the mother.

## 5 RECOMMENDATIONS

Emerging infectious diseases will continue to threaten global health, therefore, to combat and limit the effects of infectious diseases, efforts from public health agencies, government, health workers, and citizens are required. That is, it should be the responsibility of all.

### 5.1 Monitoring and banning of large social gatherings

Considering the nature of transmission of this infection, the primary factors (social gatherings and congestion) should either be banned, regulated, or monitored depending on the disease condition or rate of spread and transmission. This is because, in gatherings such as recreation centers, playgrounds, and other religious activities, the virus can be transmitted between infected and susceptible children‐infection can spread using nappies, touching unclean surfaces, as well as putting things directly into the mouth.

### 5.2 Provision of online studying platforms for children

As the virus is most susceptible to children less than 5 years old, the government should make a provision with both private and public schools to transform their method of teaching to online for this period. This will assist to reduce the gathering of these children and keep them safe with their parents.

### 5.3 Sensitization of the public and health workers about the disease

Effective education is required in dealing with this infection. Educating health workers about their roles in infectious disease responses, and public awareness on how to prevent and control the diseases should be a major concern. The school and home teachers should be educated as well because the children spend 4–10 h in school. This means that there is the possibility of the children getting infected in school if they do not adhere to public health measures created in school to reduce the disease.

### 5.4 Strengthening of surveillance networks

It is also important to strengthen disease surveillance in unaffected areas, screening individuals especially travelers in airports, train stations, assemblies, or conventions. So also, environments, for example, soil and water bodies are habitats of many infectious agents that should be scrutinized routinely to detect new pathogens.

### 5.5 Policy decisions

Policymakers involved in public health should ensure that policies are made, and the government machinery should ensure that the citizens ensure a barrier is set between infectious agents and the population.

## 6 CONCLUSION

The outbreak of Tomato flu has caused many worries among parents and the Indian government. The State governments are providing all the necessary needs to reduce the spread of the virus, but more attention needs to be done to completely eradicate the virus. All the possible solutions discussed in this article are required to tangle the present challenges that are being faced to reduce the outbreak. We must know that our children are the leaders of tomorrow. Containing this outbreak should be our priority to make our children the leaders.

## AUTHOR CONTRIBUTIONS

**Ridwan Olamilekan Adesola**: Conceptualization; data curation; methodology; writing—original draft; writing—review and editing. **Ibrahim Idris**: Conceptualization; data curation; writing—original draft; writing—review and editing. **Emmanuel Opuni**: Data curation; writing—original draft; writing—review and editing. **Joanna Nicole D'Souza**: Conceptualization; data curation; writing—original draft; writing—review and editing.

## CONFLICT OF INTEREST STATEMENT

The authors declare no conflict of interest.

## TRANSPARENCY STATEMENT

The lead author Joanna Nicole D'Souza affirms that this manuscript is an honest, accurate, and transparent account of the study being reported; that no important aspects of the study have been omitted; and that any discrepancies from the study as planned (and, if relevant, registered) have been explained.

## Data Availability

The authors have nothing to report.
